# On Breaking and Bending of Lithotritic Instruments

**Published:** 1846-11

**Authors:** 


*On breaking and bending of Lithotritic Instruments.* By M. Civiale.—M. Civiale, after noticing the recorded cases of those accidents, considers what is the practice that should be adopted in such cases. When a portion of the instrument is completely detached and falls into the bladder, the event is by no means so formidable as when the instrument is only partially broken. In the former case the patient sustains no immediate suffering—immediate interference is not essential, and as may be indicated by circumstances, attempts may be made to extract the metallic fragment, or the operation of lithotomy may be performed at a fitting opportunity. But when the instrument is but partially broken, when the bent lithotrite cannot be withdrawn from the bladder, immediate interference is necessary; and surgeons have hitherto generally endeavoured to withdraw the instrument where the urethra has been lacerated or contused. M. Civiale in such cases recommends that the high operation for the stone should at once be performed, the surgeon having of course previously furnished himself with the instrument requisite to remedy the distortion of the lithotrite that prevents its being withdrawn, and then any bending may be corrected, a half-severed fragment may be entirely detached, or, if needful, the portion of the lithotrite in the bladder may be sawn off. The lithotrite itself being in the bladder will facilitate the performance of the operation.

M. Civiale strongly deprecates, as not only useless but injurious, attempts to withdraw the lithotrite, (which have been so frequently made by surgeons under such circumstances,) ignorant as we must of necessity be as to the nature and situation of the obstacle. Even when the instrument is brought into the membranous portion of the urethra, it may be utterly impossible to withdraw it further, as other parts of the urethra are not equally dilatable. He also condemns cutting down on the lithotrite in the perineum, as the bent part of the instrument may not be thus attainable. M. Civiale recommends that, in addition to the surgeon always himself testing the strength of lithotritic instruments before operating with them, the instrument should be so constructed that the surgeon cannot exert more that a certain moderate degree of force with it—an object easily effected by adjusting the power and construction.—*Ibid, from Bulletin de Therapeutique.*

